# Overall Memory Impairment Identification with Mathematical Modeling of the CVLT-II Learning Curve in Multiple Sclerosis

**DOI:** 10.1155/2012/312503

**Published:** 2012-04-29

**Authors:** Igor I. Stepanov, Charles I. Abramson, Marietta Hoogs, Ralph H. B. Benedict

**Affiliations:** ^1^Department of Neuropharmacology, Institute for Experimental Medicine, The Russian Academy of Medical Sciences, Acad. Pavlov Street 12, 197376 St. Petersburg, Russia; ^2^Department of Psychology, Oklahoma State University, 116 N. Murray, Stillwater, OK 74078, USA; ^3^The Jacobs Neurological Institute, Buffalo General Hospital, 100 High Street, Buffalo, NY 14203, USA; ^4^Department of Neurology, State University of New York (SUNY) at Buffalo, Buffalo General Hospital, Suite D6, 100 High Street, Buffalo, NY 14203, USA

## Abstract

The CVLT-II provides standardized scores for each of the List A five learning trials, so that the clinician can compare the patient's raw trials 1–5 scores with standardized ones. However, frequently, a patient's raw scores fluctuate making a proper interpretation difficult. The CVLT-II does not offer any other methods for classifying a patient's learning and memory status on the background of the learning curve. The main objective of this research is to illustrate that discriminant analysis provides an accurate assessment of the learning curve, if suitable predictor variables are selected. Normal controls were ninety-eight healthy volunteers (78 females and 20 males). A group of MS patients included 365 patients (266 females and 99 males) with clinically defined multiple sclerosis. We show that the best predictor variables are coefficients *B3* and *B4* of our mathematical model *B3* ∗ exp(−*B2*  ∗  (*X* − 1)) + *B4*  ∗  (1 − exp(−*B2*  ∗  (*X* − 1))) because discriminant functions, calculated separately for *B3* and *B4*, allow nearly 100% correct classification. These predictors allow identification of separate impairment of readiness to learn or ability to learn, or both.

## 1. Introduction

Multiple sclerosis (MS) is an autoimmune disease of the central nervous system leading to demyelization and scarring within the cerebrum and the spinal cord [1, 2] as well as gray matter atrophy [3]. Cognitive impairment is among the main symptoms, affecting about half of all MS patients, and lower processing speed and defective retrieval from recent memory storage are frequently observed [4–6]. Impairment of cognitive functioning is correlated with brain atrophy revealed with MRI [7, 8]. Memory has multiple regional atrophy correlates, including deep gray matter [9], cerebral cortex volume [8], and medial temporal lobe volume [10, 11]. Therefore, it is reasonable to hypothesize that MS might impair attention-mediated or executive aspects such as encoding and retrieval as well as consolidation and perhaps even recognition in some circumstances [11, 12]. That is why we hypothesize that depending on the affected MS brain region, short-term memory or long-term memory might be worsened predominantly and independently.

The California Verbal Learning Test: Second Edition (CVLT-II) [13], which is the revised version of the test that was initially published in 1987 [14], was selected by a group of neuropsychologists and neurologists to assess auditory/verbal memory defects in MS [15]. Benedict and colleagues [16] recently confirmed the validity of the CVLT-II with a large cohort of MS data. Of the 23 measures assessed, 18 significantly discriminated MS patients from demographically matched controls at the conventional *P* < .05 threshold, and 14 measures discriminated the groups at *P* < .001. The CVLT-II was able to distinguish between groups of healthy participants and MS patients. A discriminant function analysis of the data retained five measures: short delay free recall, recognition discrimination index, long delay free recall, semantic clustering, retroactive interference; with 70.0% of the participants correctly classified [16]. In addition to the conventional CVLT-II measures available from the test manual and its companion computer program, Stegen and coauthors [16] used learning measures derived from a mathematical model based on the first-order transfer function for the assessment of the learning curve [17, 18]. The model's measures were valid in combination with some other CVLT-II standard measures in discriminating MS patients from demographically matched controls. However, Stegen and colleagues did not study whether modeling of the learning curve of the List A could be used to correctly classify between groups of healthy participants and MS patients.

Though the CVLT-II provides comprehensive analysis of short- and long-term memory status, administration of the standard CVLT-II form takes 30 minutes testing with a 20-minute delay interval [19]. If examination time is limited and a neuropsychologist requires less detailed information, then a quick method for assessing memory impairment in MS patients is needed. Delis and colleagues developed the California Verbal Learning Test-II Short Form (CVLT-II SF) that was designed for two purposes: (1) as a cost-effective screening tool to identify memory problems and (2) to assess verbal learning and memory without overly taxing patients [13]. However, the CVLT-II SF includes a list of nine words instead of sixteen. In our opinion, nine words are not enough for an accurate assessment of long-term memory because many individuals are able to learn and recall 15-16 words [16].

To assess both short-term and long-term memory, many MS researchers still rely on the predictive validity of CVLT-II learning trials 1–5 because on the one hand, it is part of a consensus battery [15] and, on the other, it is useful as a standalone free recall test for diagnosis of overall memory impairment. The latter feature attracts attention because the time required for clinical assessments of MS patients is important from both an economic standpoint and avoiding patient fatigue. Presentation of List A without the subsequent subtests (learning of the list B, delay trials, etc.) during initial examination of a patient lessens the burden on the patient. If List A does not reveal significant overall memory impairment, then additional memory testing for the patient under examination may not be required.

The CVLT-II provides standardized scores for each of the five learning trials, so that the clinician can assess the consistency of the patient's immediate-recall performance across trials. The consistency means that the number of correct recalled words increases more or less monotonously from trial to trial. However, frequently fluctuating emotional state, incomplete effort, medication effects, pain symptoms, and so forth influence the raw scores. Thus, poor scores on one or two trials in the face of better recall on the other trials can lead to an inaccurate assessment of learning and memory.

In this paper, we advocate discriminant analysis in the evaluation of learning curves. The discriminant analysis creates a linear combination of predictor variables that provides the best discrimination between, for example, a control group of healthy individuals and a group of MS patients [20]. If the raw scores trials 1–5 are used as predictor variables, discriminant analysis outputs a multidimensional linear function *y* = *C*_0_ + *C*_1_ ∗ Trial  1 + *C*_2_ ∗ Trial  2 + *C*_3_ ∗ Trial  3 + *C*_4_ ∗ Trial  4 + *C*_5_ ∗ Trial  5 and a boundary value for this function *Y*_*b*_. A clinician substitutes predictor variables—Trial 1, Trial 2, and so forth with raw scores trials 1–5—and calculates a value of the discriminant function *y*. If *y* ≥ *Y*_*b*_, there is no memory impairment in the patient. If *y* < *Y*_*b*_, then the patient needs detailed clinical examination to find the major cause of memory impairment. Thus, a relevant question arises: what measures of the learning curve should be used as predictor variables for discriminant analysis. In our opinion, mathematical modeling of List A learning curve provides two coefficients—*B*3 and *B*4 (see below) that are better predictors than standard CVLT-II measures Trials 1–5.

**Description of Our Mathematical Model**

Control theory defines the general transfer function as follows [21, 22]. If we designate an input signal acting upon the system as *F* and the output signal (the reaction of the system)—as *y*, then the transfer function [1/*K*] = *y*/*F*. If the input signal is equal to zero at time *t* < 0 and is equal to *F* at *t* ≥ 0, it is called “the step function.” Reaction of the first-order system on the step function is described with the differential equation *τ* · *dy*/*dt* + *y* = *F*/*K*. The equation can be rewritten in the form *dy*/*dt* = (1/*τ*) · (*F*/*K* − *y*). Define *y*_ss_ ≡ *F*/*K*. This is the asymptotic (steady) value of the output signal at *t* = *∞*. The time constant of the system is defined as *τ*. Define *a* ≡ 1/*τ*. The differential equation takes the form *dy*/*dt* = *a*  (*y*_ss_ − *y*). Thus, it is seen that the rate of the transient process in the first-order system is proportional to the difference between the asymptotic and current value of the system's output signal. The solution of the equation with initial condition *y* = *y*_0_ at *t* = 0 gives the function of exponential type *y* = (*y*_0_ − *y*_ss_)*e* ^−*at*^ + *y*_ss_. The measure of the rate of the transient process is the time constant (*τ*) that reveals how much time is necessary for achievement of 63% from the difference between the initial (*y*_0_) and asymptotic (*y*_ss_) levels of the output signal [21, 22].

We adopted the first-order transfer function for modeling the learning curves in the form *B*3∗exp⁡⁡(−*B*2∗(*X* − 1)) + *B*4∗(1 − exp⁡⁡(−*B*2∗(*X* − 1))). *X* is the trial number and *Y* is the quantity of correctly recalled words without repetitions. The parameters are *B*2—the learning rate; *B*3—the value of correctly recalled words on the first trial (i.e., *B*3 = *Y* at *X* = 1); *B*4—the asymptotic value of recalled words at *X* = *∞*.

Using the independent variable in the form of (*X* − 1) means that *B*3 is very close to the CVLT-II Trial 1 standard measure. The number of correct recalled words on the first trial assesses mainly short-term memory volume [23]. Delis and colleagues have indicated that “performance on the first immediate-recall trial of List A (Trial 1) is thought to be especially dependent on auditory attention span” [13, page 28]. Factors such as motivation [24], general health, and previous experience with the test might also influence the first trial recall. Thus, we treat *B*3 as an estimator of the general functional state of a participant before starting the test and call it “readiness to learn” [18], hence *B*3 is an abbreviation for “readiness to learn.” In our model, *B*3 primarily represents attention span and short-term memory encoding process.

Learning in either version of the CVLT begins when the list of words is presented repeatedly with trial 2 being the first repetition. As the developers of the CVLT-II indicate, “When neurologically intact individuals are provided with additional opportunities to learn a “supraspan” list of words (i.e., a list of words longer than  7 ± 2  words), their recall on the subsequent trials typically exceeds their auditory attention span and increases with each new trial.” Thus, encoding into and retrieval from LTM plays an increasingly greater role in recall performance with each representation of the same list. Performances on List A Trials 2 to 5 of the CVLT-II reflect the core verbal learning abilities of the examinee" [13, page 29]. Thus, in our model, *B*4 predominantly assesses long-term memory. Other factors such as fluctuating emotional state, medication effects, pain symptoms, and effort also influence *B*4 values [13]. We treat *B*4 as an estimator of general ability to learn and call it “ability to learn” [18]; hence, *B*4 is an abbreviation for “ability to learn”. In our model, *B*4 is a measure of long-term memory.

Velocity of learning is characterized by the time constant that reveals how much trials (after trial 1) are necessary for achieving 63% from the difference between *B*3 and *B*4. Coefficient *B*2 is inverse value of the time constant, so that the higher the value of *B*2 the faster is the learning rate. Hence, *B*2 is an abbreviation for “the learning rate.” The CVLT-II provides a measure of the learning rate in the form of the learning slope score that is calculated by a least squares regression of the linear model and reflects the average number of newly recalled words per trial. The learning slope has an important shortcoming. If a person does very well on trial 1, there tends to be a ceiling effect with little room for a learning slope. The slope is steep when a person does less well on Trial 1 and then does better in subsequent trials. In such a situation, the CVLT-II learning slope is misleading when a person earns a high score on trial one. Generally, from mathematical point of view, there might be a correlation between the CVLT-II learning slope and difference between maximal score over all trials (*Y*_max⁡⁡_) and a score on Trial 1 (*Y*_1_). Mathematically, learning  slope = *a*_0_ + *a*_1_∗(*Y*_max⁡⁡_ − *Y*_1_). We found that the CVLT-II learning slope significantly correlated with the difference between the maximal number of recalled words and the number of recalled words on trial 1 [25].

Our coefficient *B*2, reflecting the number of trials needed to reach difference between *B*3 and *B*4, differs in principal from the Learning Slope. The Pearson correlation between *B*2 and (*Y*_max⁡⁡_ − *Y*_1_) is nonsignificant [25]. In other words, *B*2 is more robust relative to a high score on trial 1. Nevertheless, we assume that, when comparing the learning rate between two people, *B*2 gives reliable information, if readiness to learn (*B*3) and ability to learn (*B*4) are the same in both people.

At this point in our model construction, the meaning of *B*2 is difficult to specify. We believe that on the whole *B*2 might reflect the rate of information transfer from short-term memory to the hippocampus and the rate at which the hippocampus can encode new episodic memories. As the construct validity of *B*2 is poorly understood, we focused on *B*3 and *B*4 as possible valid predictor variables for discriminant analysis.

Previously we applied our mathematical model to the CVLT learning curve for patients with type 2 diabetes mellitus [26] and next outlined four procedures that must be implemented to achieve nearly 100% of correct classification of learning deficits in these patients. These procedures include (1) dividing participants and patients by gender; (2) dividing participants and patients by high versus low recall with previous modeling each individual learning curve, forming samples of *B*3 and *B*4, and next clustering healthy participants and MS patients by *B*3 or *B*4; (3) conducting separate discriminant analysis of readiness to learn (coefficient *B*3) and ability to learn (coefficient *B*4); (4) comparing a sample of healthy participants with high recall scores, (or with high values of *B*3 or *B*4) with a sample from patients with low recall scores, (or with low values of *B*3 or *B*4). Fulfillment of these procedures allows calculating correct boundary values for *B*3 and *B*4 with discriminant analysis [26].

**Dividing Participants and Patients by Gender**

First and foremost, each group of participants and patients should be divided by gender because women outperform men on the CVLT [27] and girls outperform boys on the CVLT-Children's version [28]. In particular, women score significantly higher than men do on all immediate free recall trials, having recalled an average of approximately one word per trial more than men have. Women use active, semantically mediated strategies during encoding, whereas men prefer to recall words in the order in which they were presented, which is a less effective organizational strategy, and are more inclined to recall items from the primacy and recency regions [29].

The different encoding strategies among males and females have an effect on such CVLT/CVLT-II measures as the Semantic Clustering index and the Serial Clustering index. A semantic cluster occurs when two words in adjacent recall positions are members of the same category and a serial cluster occurs when two words are recalled in the same sequence in which they presented on the list [14, 30]. In a group of men and women, matched for age and education, a mean value of the semantic clustering index was 2.53 for females versus 1.94 for males (*P* < .005), but the serial clustering index was 1.86 in females versus 2.48 in males (*P* < .05), confirming that women use more efficient mode of organization of verbal information on free recall trials than men [27]. Thus, the CVLT/CVLT-II is constructed with an initial bias toward women and this bias must be taken into consideration to optimize modeling of the learning curve. In addition, gray matter and central atrophy are more advanced in male MS patients, whereas white matter atrophy is more advanced in female MS patients [31].

**Separate Assessment of *B*3 and *B*4**

Developers of the CVLT/CVLT-II/CVLT-C divide learning and memory impairment as assessed with List A into three patterns [13]. The first pattern is performed by individuals with impaired attention but normal learning and memory who may perform poorly on trial 1 but recoup and perform adequately on subsequent trials. In our terminology, this pattern stands for insufficient readiness to learn.

The second pattern may be observed in individuals with some neurological disorders that damage the memory circuitry of the brain but leave attentional processes relatively intact. These individuals perform in the average or near-average range on trial 1 but on subsequent trials fail to assimilate additional information beyond their initial attention span. In our terminology, this pattern stands for insufficient ability to learn.

The third pattern is seen in individuals with deficiencies in both attention and learning. These individuals perform poorly on trial 1 and continue to do so on subsequent immediate-recall trials. In our terminology, this pattern stands for insufficient readiness and insufficient ability to learn. As Delis and colleagues state, “performance on the immediate-recall trials of the CVLT-II (i.e., the first five learning trials) usually reflects a nebulous combination of STM and LTM, which precludes precise conclusions about the relative integrity of these proposed memory systems [13, page 27].”

The purpose of this study was to assess which measures of the learning curve—the standard CVLT-II measures Trials1–5 or coefficients *B*3 and *B*4 of our mathematical model—must be used as predictor variables for discriminant analysis between healthy persons and MS patients.

## 2. Materials and Methods

### 2.1. Participants

Normal controls were ninety-eight healthy volunteers (78 females and 20 males). A group of MS patients included 365 patients (266 females and 99 males) with clinically defined multiple sclerosis [32] being part of a larger previous study [16]. Throughout the text, the phrase “healthy participants” represents normal controls, and the phrase “MS patients” represents participants with clinically defined multiple sclerosis.

All were at least 18 years of age and fluent in the English language. Exclusion criteria were (a) presence of a medical disorder other than MS affecting cognitive function, (b) psychiatric disorder [33] other than mood, personality, or behavioral change following the onset of MS, (c) drug or alcohol dependence or current abuse, (d) motor or sensory impairment that could compromise testing (e.g., corrected vision of at least 20/70), and (e) relapse or corticosteroid treatment within four weeks of assessment.

Prior to participation, all research participants provided written consent as approved by institutional review boards and consented for the electronic storage of their test data. The mean (±SD) age of MS patients was 46.3 ± 9.1 years. Patients completed 14.2 ± 2.4 years of education. Disease duration was 10.7 ± 8.0 years. The majority were Caucasian (92%) and female (*n* = 266 or 72.9%), consistent with MS population demographics [34]. Disease course was as follows: relapsing remitting (RR) *n* = 266 or 72.9%, secondary progressive (SP) *n* = 80 or 21.9%, progressive-relapsing *n* = 9 or 2.5%, primary progressive *n* = 10 or 2.7%.

Healthy controls with a mean age of 45.33 ± 7.3 and 14.6 ± 1.4 years of education were recruited through newspaper advertisements, which targeted demographics consistent with the patient sample. The majority of the controls were Caucasian and women (*n* = 78 or 79.6%). The differences between the female versus male controls and MS patients in age or educational level did not reach significance using ANOVA or chi-squared tests. Other details are available elsewhere [16].

### 2.2. Procedure

A combined sample of MS patients seen for research (*n* = 91, 24.9%), routine clinical monitoring (*n* = 151, 41.4%), or clinical indication (*n* = 123, 33.7%) was used. Research participants (including all normal controls) were contacted by mail or were approached during the course of their usual clinical care at an MS center. Clinical patients were evaluated for either routine monitoring of cognitive function or for specific clinical reasons. Participants were assessed using the entire MACFIMS battery (the Minimal Assessment of Cognitive Function in Multiple Sclerosis) administered individually by a psychologist, trained assistant, or a graduate student under the supervision of a board-certified neuropsychologist. All participants were assessed at affiliated hospitals in association with the Department of Neurology at the State University of New York at Buffalo.

### 2.3. Measures

Representing the auditory/verbal memory domain, the CVLT-II was administered in the standard manner [13] as part of the Minimal Assessment of Cognitive Function in multiple sclerosis protocol [15]. The CVLT-II assesses memory with two 16-item word lists. The words are grouped into four semantic categories of four words each. In general, participants are asked to recall the first list (List A) following each of five exposures. All details on CVLT-II standard measures are available elsewhere [16]. For the purposes of the quantitative analysis of the learning curve, free recall of 16 words from list A during five consecutive trials was used [13].

### 2.4. Estimation of the Parameters of the Model and Its Verification

SPSS and Mathematica were used to estimate the model's parameters. *R* squared (*R* ^2^) was used for verification. The closer the learning data are to the model values the higher is *R* ^2^, its maximal value being equal to 1. A step-by-step guide to make the calculations using SPSS and Mathematica is provided in our recent papers [18, 25, 26]. To make the calculations as easy as possible we have, developed software (“The Learning Curve Modeling Tool”).

### 2.5. Standard Statistical Methods

SPSS was used for all statistical calculations. When discriminant analysis was used with trials 1–5, we checked an option “Enter independents together” to include each Trial value into calculations. Hierarchical cluster analysis based on Ward's method was used with trials 1–5 measures as variables entered together. *K*-means cluster analysis, which in our case is reduced to two means, was used with *B*3 or *B*4 as a single variable.

## 3. Results

### 3.1. Discriminant Analysis with Standard CVLT-II Measures Trials 1–5

To assess whether the mathematical modeling of the learning curve based on our model is more sensitive in detecting memory deficits than standard CVLT-II measures, we first calculated mean values for standard CVLT-II trials 1–5 for each trial (98 subjects). Means **(± **SEM) for the healthy participant group are presented in Table 1, item 1. Means for the MS patient group (365 subjects) are presented in the Table 1, item 2. These means were compared for each trial with independent sample *t*-test. Significant difference was found for each trial (*P* < .001). Thus, standard CVLT-II measures—trials 1–5—were used for discriminant analysis to assess whether the measures could distinguish healthy participants from MS patients.

#### 3.1.1. Discriminant Analysis for Healthy Participant and MS Patient Groups

Wilk's lambda was significant, *λ* = .894, *χ* ^2^ = 51.25, *P* < .001, indicating that the linear discriminant function based on raw free recall scores was able to significantly discriminate between healthy participants and MS patients. A boundary value for the discriminant function is  .420. Discriminant function yielded the following coefficients: *y* = −4, 774 + 0.1564 ∗ Trial  1 − 0.040 ∗ Trial  2 + 0.047∗Trial  3 − 0.04906∗Trial  4 + 0.349∗Trial  5. The discriminant function correctly predicted 80.6% of healthy participants and 61.9% of MS patients.

Taking into account that females outperform males on CVLT [27, 28], we compared each standard CVLT-II measures—trials 1–5 between males and females. The means (±S.E.M.) for the group of healthy male participants (20 subjects) are presented in Table 1, item 3. The means for female participants (78 subjects) are presented in Table 1, item 4. These means were compared for each trial with an independent sample *t*-test. Mean value for each trial is higher in females (*P* ≤ .001). The means (±S.E.M.) of male MS patients (99 subjects) are presented in Table 1, item 5. The means for female patients (266 subjects) are presented in Table 1, item 6. Mean value of each trial is higher in females (*P* ≤ .002). These results confirmed that healthy and MS females outperform males in the number of correctly recalled words from the CVLT-II List A. Therefore, we performed discriminant analysis for males and females separately.

#### 3.1.2. Discriminant Analysis for Males

 Wilk's lambda was significant, *λ* = .893, *χ* ^2^ = 12.996, *P* = .023, indicating that the linear discriminant function based on raw free recall scores was able to significantly discriminate between healthy male participants and MS male patients. A boundary value for the discriminant function is  .305. Discriminant function yielded the following coefficients: *y* = −2.666 − 0.104∗Trial  1 − 0.245∗Trial  2 + 0.295∗Trial  3 − 0.306∗Trial  4 + 0.523∗Trial  5. The discriminant function correctly predicted 70% of healthy male participants and 61.6% of MS male patients.

#### 3.1.3. Discriminant Analysis for Females

Wilk's lambda was significant, *λ* = .878, *χ* ^2^ = 44.064, *P* < .001, indicating that linear discriminant function based on raw free recall scores was able to significantly discriminate between healthy female participants and MS female patients. The boundary value for the discriminant function is  .242. The discriminant function yielded the following coefficients: *y* = −5.378 + 0.20∗Trial  1 + 0.245∗Trial  2 − 0.018∗Trial  3 + 0.011∗Trial  4 + 0.319∗Trial  5. The results show that the discriminant function correctly predicted 78.2% of healthy female participants and 62.8% of MS female patients.

We compared each standard CVLT-II measures—trials 1–5 between healthy participants and MS patients with independent sample *t*-test. In healthy males, mean values were significantly higher in comparison with MS male patients on Trial 3 (*P* = .031) and Trial 5 (*P* = .006). In healthy females, mean values were significantly higher in comparison with MS male patients on each trial (*P* ≤ .001). Nevertheless, discriminant analysis based on standard measures—trials 1–5—could classify less than 70%, when all participants within a group are used for discriminant analysis.

To increase discrimination capacity, we further separated each group (male and female healthy participants and MS patients) into two samples—one sample with high and the other sample with low levels of CVLT-II recall scores using cluster analysis.

### 3.2. Cluster Analysis of Healthy Participants and MS Patients Using Standard CVLT-II Measures Trials 1–5

#### 3.2.1. Cluster Analysis of Healthy Male Participants

**Figure 1:**
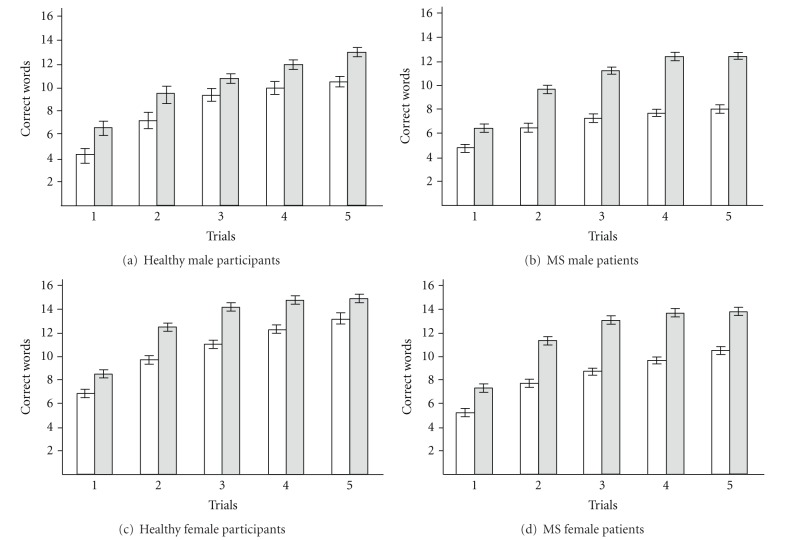
Cluster analysis of the CVLT-II measures trials 1–5. (a) Healthy male participants, cluster referring to lower memory functioning (9 cases) and cluster referring to higher memory functioning (11 cases). Comparing the values for every trial with a *t*-test for independent samples revealed that the same trial values differed between cluster referring to lower memory functioning and cluster referring to higher memory functioning (*P* < .05) with the exception of Trial 3 (*P* = .075). (b) MS male patients, cluster referring to lower memory functioning (46 cases) and cluster referring to higher memory functioning (53 cases). Comparing the values for each of the five trials with a *t*-test for independent samples, revealed significant differences between cluster referring to lower memory functioning and cluster referring to higher memory functioning for each trial (*P* ≤ .001). (c) Healthy female participants, cluster referring to lower memory functioning (38 participants) and cluster referring to higher memory functioning (40 participants). Comparing values for every trial with a *t*-test for independent samples revealed that the same trial values differed between cluster referring to lower memory functioning and cluster referring to higher memory functioning (*P* < .001). (d) MS female patients, cluster referring to lower memory functioning (133 patients) and cluster referring to higher memory functioning (133 patients). Comparing values for every trial with a *t*-test for independent samples revealed that the same trial values differed between cluster referring to lower memory functioning and cluster referring to higher memory functioning (*P* < .001). Cluster referring to lower memory functioning—white bar, cluster referring to higher memory functioning—gray bar. Each bar represents mean ± S.E.M.

The entire group was divided into two clusters: cluster referring to lower memory functioning (9 cases) with smaller values, and cluster referring to higher memory functioning (11 cases) with larger values (Figure 1(a)). Mean values (±S.E.M.) for the cluster of lower memory functioning are presented in Table 1, item 7; values for the cluster of higher memory functioning are presented in Table 1, item 8. Comparing means with a *t*-test for independent samples revealed that the cluster of higher memory functioning means were higher in comparison with the cluster of lower memory functioning for each trial (*P* < .05) with the exception of Trial 3 (*P* = .075). Thus, cluster associated with higher memory functioning better characterized the healthy male sample and was used for further discriminant analysis.

#### 3.2.2. Cluster Analysis of MS Male Patients

The entire group was divided into two clusters: one referring to lower memory functioning (46 cases) with smaller values and another referring to higher memory functioning (53 cases) with larger values (Figure 1(b)). Mean values (±S.E.M.) of the cluster representing lower memory functioning are presented in Table 1, item 9 and for cluster referring to higher memory functioning in Table 1, item 10. Comparing means with independent sample *t*-tests revealed that clusters of higher memory functioning were higher in comparison with cluster referring to lower memory functioning (*P* ≤ .001). The cluster of lower memory functioning better characterized MS male patients and was used for further discriminant analysis.

#### 3.2.3. Cluster Analysis of Healthy Female Participants

The entire group was divided into two clusters: cluster referring to lower memory functioning (38 cases) with smaller values and the other referring to higher memory functioning (40 cases) with larger values (Figure 1(c)). Mean values (±S.E.M.) for the cluster of lower memory functioning are presented in Table 1, item 11 and for cluster of higher memory functioning in Table 1, item 12. Comparing means with a *t*-test for independent samples revealed that the clusters of higher memory functioning were higher in comparison with the cluster of lower memory functioning for each trial (*P* ≤ .001). The cluster of higher memory functioning better characterized healthy female participants and was used for further discriminant analysis.

#### 3.2.4. Cluster Analysis of MS Female Patients

The entire group was divided into two clusters: one referring to lower memory functioning (133 cases) with smaller values and another, referring to higher memory functioning (133 cases) with larger values (Figure 1(d)). Mean values (±S.E.M.) for cluster of lower memory functioning are presented in Table 1, item 13 and for the cluster of higher memory functioning presented in Table 1, item 14. Comparing means with a *t*-test for independent samples revealed that the cluster of higher memory functioning means were higher in comparison with the cluster of lower memory functioning for each trial (*P* ≤ .001). Thus, cluster of lower memory functioning better characterized MS female participants sample and was used for further discriminant analysis.

### 3.3. Discriminant Analysis of Trials 1–5 Clusters Referring to Lower and Higher Memory Functioning

#### 3.3.1. Discriminant Analysis for Male Groups Clusters

The cluster of higher memory functioning with high recall raw scores from healthy male participants and the cluster of lower memory functioning with low recall raw scores from MS male patients were used for discriminant analysis. Wilks' lambda *λ* = .421, *χ* ^2^ = 45.4, *P* < .001 indicated that the linear discriminant function was able to significantly discriminate between healthy male participants and MS male patients. A boundary value for the discriminant function is 1.459. Discriminant function yielded the following coefficients: *y* = −5.123 − 0.16∗Trial  1 + 0.18∗Trial  2 + 0.186∗Trial  3 + 0.115∗Trial  4 + 0.287∗Trial  5. The discriminant function correctly predicted 100% of healthy male participants and 100% of male MS patients. Verification of the cluster of lower memory functioning from healthy males revealed that 6 out of 9 cases were classified as healthy, that is, 66.7% were correctly classified. Verification of the cluster referring to higher memory functioning from MS male patients revealed that 47 out of 53, that is, 88.7% cases were classified as healthy.

#### 3.3.2. Discriminant Analysis for Female Groups Clusters

The cluster of higher memory functioning with high recall raw scores from healthy female participants and the cluster referring to lower memory functioning with low recall raw scores from MS female patients were used for discriminant analysis. Wilks' lambda *λ* = .282, *χ* ^2^ = 213, *P* < .001, indicating that linear discriminant function was able to significantly discriminate between healthy female participants and MS female patients. A boundary value for the discriminant function is 1.881. Discriminant function yielded the following coefficients: *y* = −7.326 + 0.121∗Trial  1 + 0.191∗Trial  2 + 0.295∗Trial  3 + 0.114∗Trial  4 + 0.063 ∗ Trial  5. The discriminant function correctly predicted 100% of healthy female participants and 100% of MS female patients. Verification of the cluster of lower memory functioning from healthy females revealed that only one out of 38 cases was classified as healthy. Verification of the cluster of higher memory functioning from MS female patients revealed that 72 out of 133, that is, 54.1% cases were classified as healthy and 61 cases, that is, 45.9% were classified as MS female patients.

In summary, first dividing participants and patients by gender and then further dividing them by high versus low recall using standard CVLT-II measures trials 1–5 substantially improves classification of memory impairment in patients with multiple sclerosis. However, the use of clusters is not able to identify clearly short-term or long-term memory impairment. In the following section, we tested the ability of our model to identify short-term and long-term memory impairment in MS patients.

#### 3.3.3. The Averaged Learning Curves of the Trials 1–5 Clusters Used for Discriminant Analysis

Our mathematical model allows separate assessment of short-term and long-term memory, because *B*3 represents attention span and short-term memory encoding process, whereas *B*4 represents long-term memory consolidation process. Therefore, we modeled the learning curve for the cluster of lower memory functioning and the cluster of higher memory functioning from male and female healthy participants and MS patients.

The learning curves averaged from healthy male participants outputted the following values for the model's coefficients: for the cluster referring to lower memory functioning *B*3 = 4.39 ± 0.29, *B*4 = 11.69 ± 0.55, and for the cluster referring to higher memory functioning *B*3 = 6.90 ± 0.27, *B*4 = 15.0 ± 1.0. Comparing coefficients revealed that *B*3 and *B*4 differed (*P* = .003 and *P* = .044 accordingly). The learning curves averaged over each cluster from MS male patients outputted the following values for the coefficients: for cluster referring to lower memory functioning *B*3 = 4.80 ± 0.08, *B*4 = 8.29 ± 0.14, and for cluster referring to higher memory functioning *B*3 = 6.40 ± 0.21, *B*4 = 13.10 ± 0.34. Comparing the coefficients revealed that *B*3 and *B*4 differed (*P* = .002 and *P* = .0002 accordingly).

The learning curves from healthy female participants outputted the following values for the model's coefficients: for the cluster referring to lower memory functioning *B*3 = 6.90 ± 0.26, *B*4 = 14.92 ± 1.11, and for the cluster referring to higher memory functioning *B*3 = 8.34 ± 0.05, *B*4 = 15.01 ± 0.04. Comparing of coefficients revealed that *B*3 differed (*P* = .006), but *B*4 did not differ (*P* > .2). Here, clustering with standard measures—trials 1–5 separated individual cases only by *B*3, but not *B*4. The learning curves averaged over each cluster from MS female patients outputted the following values for the model's coefficients: for the cluster referring to lower memory functioning *B*3 = 5.36 ± 0.17, *B*4 = 10.99 ± 0.44, and for the cluster referring to higher memory functioning *B*3 = 7.34 ± 0.08, *B*4 = 14.22 ± 0.09. Comparing the coefficients revealed that *B*3 and *B*4 differed (*P* = .0005, and *P* = .002 accordingly).

**Figure 2:**
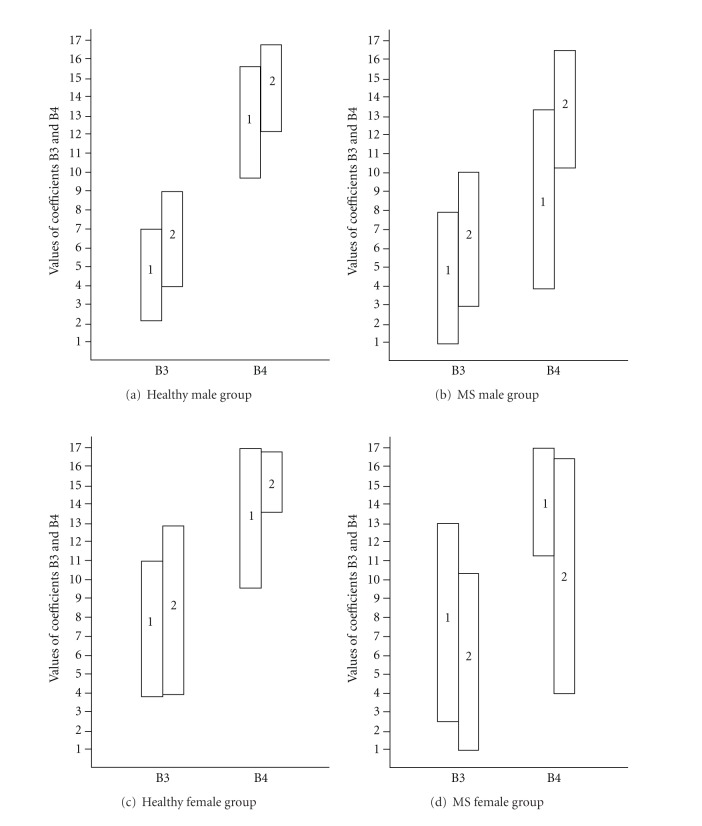
Overlapping of *B*3 or *B*4 values between clusters after clustering CVLT-II measures trials 1–5. (a) Healthy male group; (b) MS male group; (c) Healthy female group; (d) MS female group. 1 is the range of the cluster referring to lower memory functioning; 2 is the range of the cluster referring to higher memory functioning. Identification of coefficients is given on the abscissa. This figure illustrates that clustering of raw recall scores does not allow distinguishing between *B*3 and *B*4 due to considerable overlapping of each coefficient's values between cluster referring to lower memory functioning and cluster referring to higher memory functioning from healthy participants and MS patients.

Our results suggest that clustering with standard measures—trials 1–5 separated subjects by *B*3 and *B*4.  Figure 2 illustrates that clustering of raw recall scores did not distinguish between *B*3 and *B*4 due to considerable overlap between the cluster of lower memory functioning and the cluster of higher memory functioning in healthy participants and MS patients. This overlap suggests the possibility of obtaining misleading information on the level of readiness to learn and ability to learn. Thus, modeling of the learning curve might be necessary to distinguish between short-term and long-term impairment in MS patients.

### 3.4. Use of Coefficients *B*3 and *B*4 as Predictor Variables for Discriminant Analysis

In the next sections, we present results of the use of coefficient *B*3 and *B*4 for discriminant analysis.

#### 3.4.1. The Averaged Learning Curve of Healthy Participants

**Figure 3:**
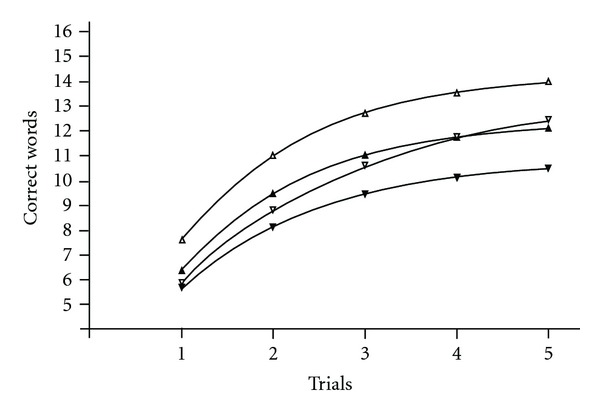
The averaged learning curves over all healthy participants and MS patients. Empty triangles—Healthy female participants; *B*2 = 0.70; *B*3 = 7.63; *B*4 = 14.38. Filled triangles—MS female patients; *B*2 = 0.72; *B*3 = 6.35; *B*4 = 12.44. Empty down triangles—Healthy male participants; *B*2 = 0.47; *B*3 = 5.90; *B*4 = 13.58. Filled down triangles—MS male patients; *B*2 = 0.64; *B*3 = 5.66; *B*4 = 10.86. *B*3 is higher in healthy females versus healthy males (*P* = .0016). *B*3 (*P* = .0019) and *B*4 (*P* = .0018) is higher in healthy female participants versus MS female patients. *B*4 is higher in healthy male participants versus MS male participants (*P* = .006). *B*3 (*P* = .006) and *B*4 (*P* = .002) is higher in MS female patients in comparison with MS male patients.

The averaged learning curve of all healthy participants (Table 2, item 1), the 78 female participants (Table 2, item 2), and the 20 male participants (Table 2, item 3) provided good fits with our model based on very high values of *R*-squared. Comparison of the coefficients revealed that *B*3 was higher in females (*P* = .0016). Other coefficients did not differ (*P* = .075 for *B*2 and *P* = .20 for *B*4). The averaged learning curve of females (Figure 3, empty triangles) was positioned above the curve for males (Figure 3, empty down triangles).

#### 3.4.2. The Averaged Learning Curve of MS Patients

The averaged learning curve sampled from MS patients also differed between men and women. The averaged curve of all MS patients (Table 2, item 4), 266 female MS patients (Table 2, item 5), and 99 male MS patients (Table 2, item 6) provided good fits with our model based on very high values of R-squared. Coefficient *B*3 (*P* = .006) and coefficient *B*4 (*P* = .002) was higher in MS female patients (Figure 3, filled triangles) in comparison with MS male patients (Figure 3, filled down triangles). The coefficient *B*2 did not differ (*P* = .32).

#### 3.4.3. Comparison of the Averaged Learning Curve of Healthy Participants and MS Patients

The averaged learning curve in MS patients differs significantly from the averaged learning curve in healthy participants both for the total learning data and for learning data of females or males calculated separately. Comparison of the averaged learning curve for the group of healthy participants (Table 2, item 1) and the group of MS patients (Table 2, item 4) revealed that *B*3 (*P* = .002) and *B*4 (*P* = .001) were higher in healthy participants though *B*2 did not differ (*P* = .56). The averaged learning curve for healthy participants was positioned nearly in parallel above the averaged learning curve for MS patients.

Separate comparison of healthy female participants (Table 2, item 2) with female MS patients (Table 2, item 5) revealed lower values of *B*3 (*P* = .0019) and *B*4 (*P* = .0018) in female MS patients with no difference between *B*2 (*P* = .88). The averaged learning curve for healthy female participants was positioned above the averaged learning curve for female MS patients (see Figure 3).

Unlike females, comparison of healthy male participants (Table 2, item 3) with male MS patients (Table 2, item 6) revealed that *B*2 (*P* = .13) and *B*3 (*P* = .29) were not affected, but *B*4 was higher in healthy male participants (*P* = .006). Both learning curves practically coincide at trial 1 (see Figure 3). Coefficient *B*3 (*P* = .006) and *B*4 (*P* = .002) was higher in MS female patients in comparison with MS male patients.

Our results supported the existence of CVLT-II gender bias in favor of females as it was previously shown for CVLT [27]. The existence of a gender bias necessitates that the discriminant and cluster analyses be performed separately for males and females, if our model is to provide greater precision with respect to identifying memory impairment in patients with multiple sclerosis and ensure close to 100% correct classification relative to other CVLT-II measures.

### 3.5. Cluster Analysis of Healthy Male Participants and MS Male Patients Using Coefficients *B*3 or *B*4

Previous to performing cluster analysis, we modeled each individual learning curve and formed samples of *B*3 and *B*4. Next, we clustered healthy participants and MS patients by *B*3 or *B*4. Then, the raw recall scores—trials 1–5 of participants and patients from each cluster—were used to calculate the averaged learning curves.

#### 3.5.1. Healthy Male Participants: Clustering Using *B*3 Only

**Figure 4:**
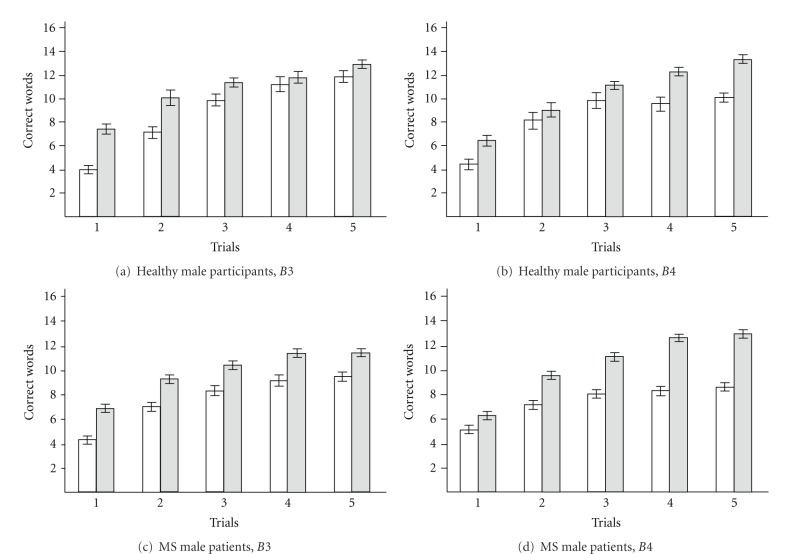
Cluster analysis using *B*3 or *B*4 in males. (a) Healthy male participants: clustering using *B*3 only, cluster referring to lower memory functioning (9 cases) and cluster referring to higher memory functioning (11 cases). There is no overlap between these *B*3 clusters. (b) Healthy male participants: clustering using *B*4 only, cluster referring to lower memory functioning (5 cases) and cluster referring to higher memory functioning (15 cases). There is no overlap between these *B*4 clusters. (c) MS male patients: clustering using *B*3 only, cluster referring to lower memory functioning (48 cases) and cluster referring to higher memory functioning (51 cases). There is no overlap between these *B*3 clusters. (d) MS male patients: clustering using *B*4 only, cluster referring to lower memory functioning (59 cases) and cluster referring to higher memory functioning (40 cases). There is no overlap between these *B*4 clusters. Cluster referring to lower memory functioning—white bars, cluster referring to higher memory functioning—gray bars. Each bar represents mean ± S.E.M.

**Figure 5:**
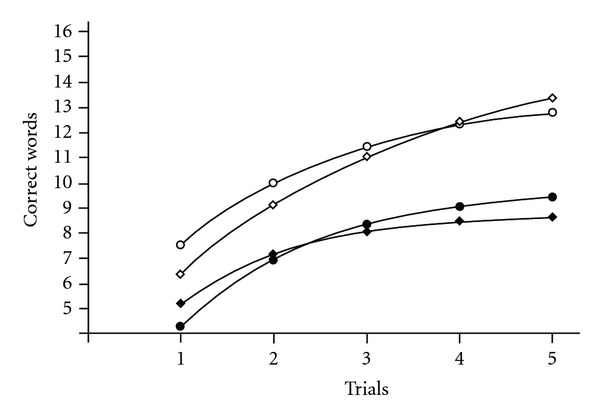
The averaged learning curves over cluster referring to lower memory functioning and cluster referring to higher memory functioning for healthy male participants and MS male patients, using *B*3 or *B*4. Empty circles—cluster referring to higher memory functioning using *B*3 in healthy male participants; *B*2 = 0.53; *B*3 = 7.50; *B*4 = 13.46. Empty diamonds—cluster referring to higher memory functioning using *B*4 in healthy male participants; *B*2 = 0.34; *B*3 = 6.39; *B*4 = 15.76. Filled circles—cluster referring to lower memory functioning using *B*3 in MS male patients; *B*2 = 0.65; *B*3 = 4.26; *B*4 = 9.81. Filled diamonds—cluster referring to lower memory functioning using *B*4 in MS male patients; *B*2 = 0.82; *B*3 = 5.17; *B*4 = 8.74. In healthy male participants, *B*3 is higher in cluster referring to higher memory functioning using *B*3 (*P* = .04) in comparison with cluster referring to higher memory functioning using *B*4. In MS patients, *B*3 was higher in cluster referring to lower memory functioning using *B*4 (*P* = .0002). However, *B*4 was higher in cluster referring to lower memory functioning using *B*3 (*P* = .0008).

Clustering using *B*3 separated all cases into two clusters: one cluster of lower memory functioning with low values of *B*3 in the range of 2.18 to 5.0 for 9 male participants and cluster referring to higher memory functioning with high values of *B*3 in the range of 5.97 to 9.0 for 11 male participants (Figure 4(a)). Values of the model's coefficients are given in Table 2, items 7 and 8. The value of *B*3 is higher (*P* = .002) in the cluster associated with higher memory functioning. The averaged learning curve for the cluster of higher memory functioning is shown in Figure 5, empty circles. The cluster of higher memory functioning values better characterized the healthy male sample and was used for further discriminant analysis.

#### 3.5.2. Healthy Male Participants: Clustering Using *B*4 Only

Clustering using *B*4 also separated all cases into two clusters: one referring to lower memory functioning with low values of *B*4 in the range 9.71 to 11.31 for 5 male participants and a cluster referring to higher memory functioning with high values of *B*4 in the range 12.24 to 16.73 for 15 male participants (Figure 4(b)). Values of the model's coefficients are in Table 2, items 9 and 10. Coefficient *B*3 and coefficient *B*4 was higher (*P* = .01 and *P* = .0007, resp.) in the cluster of higher memory functioning compared to the cluster of lower memory functioning. The averaged learning curve for higher memory functioning is shown in Figure 5, empty diamonds. Comparison of the coefficients in the higher memory cluster using *B*3 and using *B*4 revealed that *B*3 was higher (*P* = .04) in the cluster of higher memory functioning using *B*3. Thus, cluster referring to higher memory functioning values better characterized the healthy male sample and was used for further discriminant analysis.

#### 3.5.3. MS Male Patients: Clustering Using *B*3 Only

Clustering using *B*3 separated all cases into two clusters: one referring to lower memory functioning with low values of *B*3 in the range 0.99 to 5.22 for 48 MS male patients and another referring to higher memory functioning with high values of *B*3 in the range 5.64 to 10.02 for 51 MS male patients (Figure 4(c)). Values of the model's coefficients are given in Table 2, items 11 and 12. The value of *B*3 (*P* = .00006) and *B*4 (*P* = .002) was higher in the cluster referring to higher memory functioning. The averaged learning curve for the cluster referring to lower memory functioning is shown in Figure 5, filled circles. Though MS male patients were separated using *B*3, these patients also revealed low value of *B*4. Thus, cluster referring to lower memory functioning better characterized the MS male sample and was used for further discriminant analysis.

#### 3.5.4. MS Male Patients: Clustering Using *B*4 Only

Clustering using *B*4 separated all cases into two clusters: one referring to lower memory functioning with low values of *B*4 in the range of 3.95 to 12.35 for 59 MS male patients and one referring to higher memory functioning with high values of *B*4 in the range of 12.85 to 16.43 for 40 MS male patients (Figure 4(d)). Values of the model's coefficients are given in Table 2, items 13 and 14. The value of *B*3 (*P* = .013) and *B*4 (*P* = .0014) was higher in cluster of higher memory functioning. Clustering based on coefficient *B*4 separated the group of MS male patients into two different clusters. The averaged learning curve for the cluster of lower memory functioning is shown in Figure 5, filled diamonds. Though MS male patients were separated using *B*4, coefficient *B*3 was also impaired to some extent. Comparison of the coefficients between the cluster of lower memory functioning using *B*3 and *B*4 revealed that *B*3 was higher in the cluster of lower memory functioning using *B*4 (*P* = .0002). However, *B*4 was higher in the cluster of lower memory functioning using *B*3 (*P* = .0008). Thus, cluster referring to lower memory functioning better characterized the MS male sample and was used for further discriminant analysis.

### 3.6. Discriminant Analysis of the Cluster of Higher Memory Functioning for Healthy Male Participants and the Cluster of Lower Memory Functioning for MS Male Patients

Cluster analysis allowed separating healthy participants with high values of *B*3 or *B*4 and MS patients with low values of the same coefficients using *B*3 or *B*4. Both coefficients (*B*3, *P* = .0009 and *B*4, *P* = .013) differed significantly between the cluster referring to higher memory functioning for healthy males and the cluster referring to lower memory functioning for MS males using *B*3. *B*3 (*P* = .0002) and *B*4 (*P* = .0001) also differed significantly between the cluster of higher memory functioning in healthy males and the cluster of lower memory functioning for MS males using *B*4. We now use discriminant analysis on these clusters.

#### 3.6.1. Discriminant Analysis Using *B*3 Only

First, discriminant analysis was conducted to assess whether coefficient *B*3 only could distinguish the cluster of higher memory functioning for healthy male participants from the cluster of lower memory functioning for MS male patients. Wilk's lambda was significant, *λ* = .406, *χ* ^2^ = 50.958, *P* < .001, indicating that *B*3 only was able to significantly discriminate between the two groups. The boundary value for the discriminant function is  .958. Discriminant function yielded the following coefficients: *y* = −4.518 + 0.937∗*B*3. The discriminant function correctly predicts 100% of healthy participants and 100% of MS patients. The quality of the discrimination was assessed with the cluster of lower memory functioning (low *B*3 values) using *B*3 for healthy males. Discriminant function values for all 9 of 9 cases were less than 0.958. We also assessed the quality of the cluster of higher memory functioning (high *B*3 values) for MS male patients. Here, only one patient with *B*3 = 5.64 was classified as belonging to the MS group, but 50 out of 51 patients were correctly classified as belonging to the healthy group. Thus, 98.0% of participants were classified with our model correctly.

#### 3.6.2. Discriminant Analysis Using *B*4 Only

Discriminant analysis was also conducted to assess whether coefficient *B*4 only could distinguish between the cluster of higher memory functioning for healthy male participants from the cluster of lower memory functioning for MS male patients. Wilk's lambda was significant, *λ* = .534, *χ* ^2^ = 44.820, *P* < .001, indicating that the model using *B*4 only was able to successfully discriminate between the two groups. The boundary value for the discriminant function is  .681. Discriminant function yielded the following coefficients: *y* = −4.923 + 0.466∗*B*4. These results show that the discriminant function correctly predicts 100% of healthy participants (15 of 15) and 91.5% of MS patients (54 of 59). Five cases from the cluster of lower memory functioning using *B*4 for MS males with *B*4 values from 12.11 to 12.35 were classified as belonging to the healthy group. Thus, 93.2% of original grouped cases are correctly classified. The quality of the discrimination was assessed with the cluster of lower memory functioning (low *B*4 values) using *B*4 for healthy males. Discriminant function values for each of the 5 cases were less than 0.681 representing 100% correct classification. Next, the quality of the cluster of higher memory functioning (high *B*4 values) was assessed for MS male patients. All 40 of 40 patients were classified as belonging to healthy group correctly. Thus, 100% are correctly classified.

### 3.7. Cluster Analysis of Healthy Female Participants and MS Female Patients with *B*3 or *B*4

 Previous to conducting the cluster analysis, we modeled each individual learning curve and formed samples of *B*3 and *B*4. Next we clustered healthy participants and MS patients by *B*3 or *B*4. Then, the raw recall scores—trials 1–5 of participants and patients from each cluster were used to calculate the averaged learning curves.

#### 3.7.1. Healthy Female Participants: Clustering Using *B*3 Only

**Figure 6:**
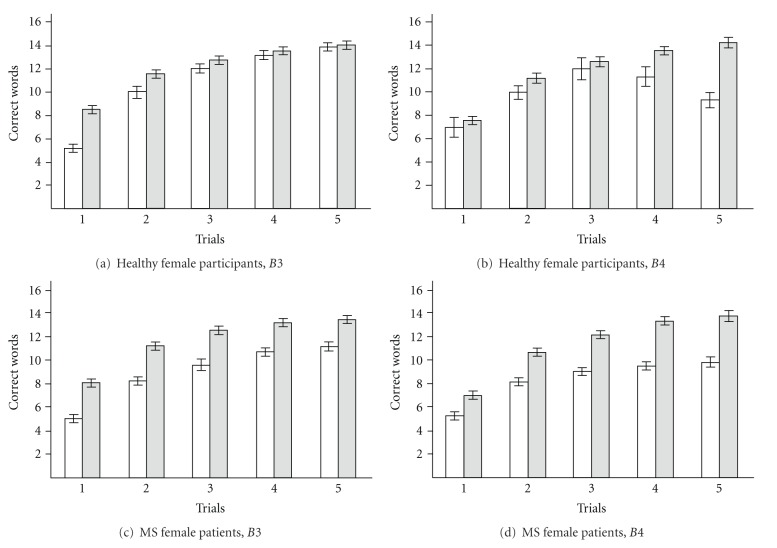
Cluster analysis using *B*3 or *B*4 in females. (a) Healthy female participants: clustering using *B*3 only, cluster referring to lower memory functioning (22 cases) and cluster referring to higher memory functioning (56 cases). There is no overlap between these *B*3 clusters. (b) Healthy female participants: clustering using *B*4 only, cluster referring to lower memory functioning (3 cases) and cluster referring to higher memory functioning (75 cases). There is no overlap between these *B*4 clusters. (c) MS female patients: clustering using *B*3 only, cluster referring to lower memory functioning (152 cases) and cluster referring to higher memory functioning (114 cases). There is no overlap between these *B*3 clusters. (d) MS female patients: clustering using *B*4 only, cluster referring to lower memory functioning (109 cases) and cluster referring to higher memory functioning (157 cases). There is no overlap between these *B*4 clusters. Cluster referring to lower memory functioning—white bars, cluster referring to higher memory functioning—gray bars. Each bar represents mean ± S.E.M.

**Figure 7:**
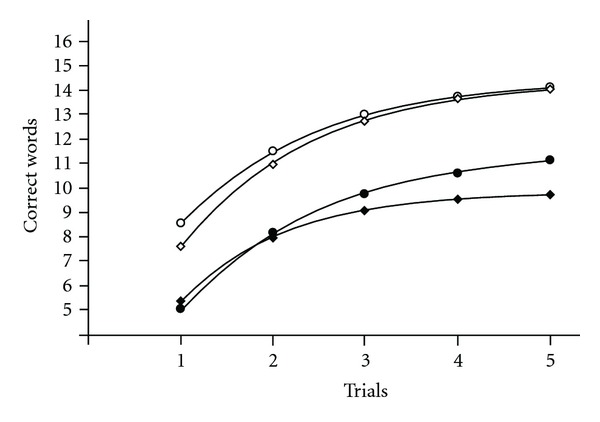
The averaged learning curves over cluster referring to lower memory functioning and cluster referring to higher memory functioning for healthy female participants and MS female patients, using *B*3 or *B*4. Empty circles—cluster referring to higher memory functioning using *B*3 in healthy female participants; *B*2 = 0.69; *B*3 = 8.56; *B*4 = 14.43. Empty diamonds—cluster referring to higher memory functioning using *B*4 in healthy female participants; *B*2 = 0.66; *B*3 = 7.67; *B*4 = 14.63. Filled circles—cluster referring to lower memory functioning using *B*3 in MS female patients; *B*2 = 0.63; *B*3 = 5.06; *B*4 = 11.62. Filled diamonds—cluster referring to lower memory functioning using *B*4 in MS female patients; *B*2 = 0.86; *B*3 = 5.41; *B*4 = 9.88. In healthy female participants *B*3 is higher in cluster referring to higher memory functioning using *B*3 (*P* = .019) in comparison with cluster referring to higher memory functioning using *B*4. In MS patients *B*4 (*P* = .003) is higher in the cluster referring to lower memory functioning using *B*3 versus cluster referring to lower memory functioning using *B*4. *B*3 (*P* = .00005) and *B*4 (*P* = .0008) differs highly significant between cluster referring to higher memory functioning for healthy females and cluster referring to lower memory functioning for MS females using *B*3. Besides *B*3 (*P* = .0006) and *B*4 (*P* = .0002) differs highly significant between cluster referring to higher memory functioning for healthy females and cluster referring to lower memory functioning for MS females using *B*4.

Clustering using *B*3 alone separated all cases into two clusters: one referring to lower memory functioning with low values of *B*3 in the range of 3.81 to 6.08 for 22 female participants and another referring to higher memory functioning with high values of *B*3 in the range of 6.8 to 12.82 for 56 female participants (Figure 6(a)). Values of the model's coefficients are in Table 2, items 15 and 16. The value of *B*3 is higher (*P* = .00025) in the cluster of higher memory functioning. Averaged learning curve for the cluster of higher memory functioning using *B*3 is shown in Figure 7, empty circles. Clustering using *B*3 separated the group of healthy female participants into two distinct *B*3 clusters. Thus, the cluster referring to higher memory functioning values better characterized the healthy female sample and was used for further discriminant analysis.

#### 3.7.2. Healthy Female Participants: Clustering Using *B*4 Only

Clustering using *B*4 alone separated all cases into two clusters: one referring to lower memory functioning with low values of *B*4 in the range of 9.58 to 11.51 for 3 female participants and another referring to higher memory functioning with high values of *B*4 in the range of 12.65 to 16.91 for 75 female participants (Figure 6(b)). Values of the model's coefficients are given in Table 2, items 17 and 18. The value of *B*4 is higher (*P* = .017) in the cluster associated with higher memory functioning. Averaged learning curve for the cluster of higher memory functioning using *B*4 is provided in Figure 3, empty diamonds. In healthy female participants, *B*3 is higher in the cluster of higher memory functioning using *B*3 (*P* = .019) in comparison with the cluster of higher memory functioning using *B*4. Thus, the cluster of higher memory functioning better characterized the healthy female sample and was used for further discriminant analysis.

#### 3.7.3. MS Female Patients: Clustering Using *B*3 Only

Clustering using *B*3 separated all cases into two clusters: one referring to lower memory functioning with low values of *B*3 in the range of 1.0 to 6.22 for 152 female participants and another referring to higher memory functioning with high values of *B*3 in the range of 6.67 to 13.0 for 114 female participants (Figure 6(c)). Values of the model's coefficients are given in Table 2, items 19 and 20. Here, clustering was performed using *B*3 alone, but nevertheless, both the *B*3 value (*P* = .00003), and the *B*4 value (*P* = .0011) was higher in the cluster referring to higher memory functioning. The averaged learning curve for the cluster of lower memory functioning using *B*3 is shown in Figure 7, filled circles. Though MS female patients were separated using *B*3, the value of *B*4 was also low. Thus, the cluster of lower memory functioning values better characterized the MS female sample and was used for further discriminant analysis.

#### 3.7.4. MS Female Patients: Clustering Using *B*4 Only

Clustering using *B*4 separated all cases into two clusters: one referring to lower memory functioning with low values of *B*4 in the range of 4.0 to 12.83 for 109 MS female patients and another referring to higher memory functioning with high values of *B*4 in the range of 12.98 to 16.99 for 157 MS female patients (Figure 6(d)). Values of the model's coefficients are given in Table 2, items 21 and 22. Despite the fact that here clustering was performed using *B*4 only, both the *B*3 value (*P* = .0005), and the *B*4 value (*P* = .00003) was higher in the cluster of higher memory functioning. The averaged learning curve for the cluster of lower memory functioning using *B*3 is provided in Figure 7, filled diamonds. Though MS female patients were separated using *B*4, the value of *B*3 was also low. Thus, the cluster of lower memory functioning better characterized the MS female sample and was used for further discriminant analysis.

### 3.8. Discriminant Analysis of the Cluster of Higher Memory Functioning for Healthy Female Participants and the Cluster of Lower Memory Functioning for MS Female Patients

Cluster analysis allowed us to separate healthy participants with high values of *B*3 or *B*4 and MS patients with low values of the same coefficients using *B*3 or *B*4. Coefficient *B*3 (*P* = .00005) and coefficient *B*4 (*P* = .0008) differed significantly between the cluster of higher memory functioning for healthy females and the cluster of lower memory functioning for MS females using *B*3. *B*3 (*P* = .0006) and *B*4 (*P* = .0002) also differed significantly between the cluster of higher memory functioning for healthy females and the cluster of lower memory functioning for MS females using *B*4. We now use discriminate analysis on these clusters.

#### 3.8.1. Discriminant Analysis Using *B*3 Only

First, discriminant analysis was conducted to assess whether coefficient *B*3 alone could distinguish the cluster of higher memory functioning for healthy female participants from the cluster of lower memory functioning for MS female patients. Wilk's lambda was significant, *λ* = .372, *χ* ^2^ = 202.964, *P* < .001, indicating that the model using *B*3 alone significantly discriminate between the two groups. The boundary value for the discriminant function is  .672. Discriminant function yielded the following coefficients: *y* = −4.923 + 0.827∗*B*3. The results show that the discriminant function correctly predicts 100% of healthy participants and 100% of MS patients. The quality of the discrimination was assessed for the cluster of lower memory functioning (low *B*3 values) using *B*3 for healthy females. The values of the discriminant function for each of the 22 cases were less than 0.672 indicating that all cases were correctly classified. Next, the quality of discrimination was assessed with the cluster of higher memory functioning (high *B*3 values) for MS female patients. Here, only three patients with *B*3 values 6.67, 6.69, and 6.76, respectively, were classified as belonging to MS group, but 111 out of 114 patients were correctly classified as belonging to the healthy group. Thus, 97.4% were correctly classified.

#### 3.8.2. Discriminant Analysis Using *B*4 Only

Discriminant analysis was conducted to assess whether coefficient *B*4 alone could distinguish the cluster of higher memory functioning for healthy female participants from the cluster of lower memory functioning for MS female patients. Wilk's lambda was significant, *λ* = .301, *χ* ^2^ = 217.890, *P* < .001, which indicated that *B*4 alone was able to significantly discriminate between the two groups. The boundary value for the discriminant function is  .285. The discriminant function yielded the following coefficients: *y* = −8.399 + 0.679∗*B*4. The results show that the discriminant function correctly predicts 98.7% of healthy participants (74 out of 75) and 99.1% of MS patients (108 out of 109). Thus, 98.9% of original grouped cases are correctly classified.

The quality of the discrimination was assessed with the cluster of lower memory functioning (low *B*4 values) using *B*4 for healthy female participants. A discriminant function value for each of the three cases was less than 0.285 indicating 100% correct classification. Next, the quality of the discrimination was assessed with the cluster of higher memory functioning (high *B*4 values) for MS female patients. All 157 of 157 patients were classified correctly as belonging to healthy group using coefficient *B*4 only.

## 4. Discussion

The identification of memory impairment in multiple sclerosis is important in clinical practice. The CVLT-II is useful for assessing auditory/verbal memory defects in MS patients [15, 16]. We focused on validation of CVLT-II learning trials—trials 1–5—because it requires less time than the full test. We found that these CVLT-II measures contained sufficient information on short-term and long-term memory status and could be used for identification of overall memory impairment in patients with multiple sclerosis. However, adequacy of discriminant analysis depends on some factors that must be taken into account.

### 4.1. Dividing Participants and Patients by Gender

Our results confirm that females outperform males in the number of recalled correct words from the CVLT-II List A. In the group of healthy participants mean value at each trial is higher in females (*P* ≤ .001). In the group of MS patients, mean value at each trial is also higher in females (*P* ≤ .002). Modeling of the learning curve revealed that readiness to learn (*B*3) was higher in healthy female participants. Moreover, both readiness to learn (*B*3) and ability to learn (*B*4) were significantly higher in MS female patients in comparison with MS male patients.

Women score significantly higher than men do on all immediate free recall trials, having recalled an average of approximately one word per trial more than men. Women use active, semantically mediated strategies during encoding, whereas men prefer to recall words in the order in which they were presented, which is a less effective organizational strategy [29].

### 4.2. Dividing Participants and Patients by High and Low Recall Scores

Our results confirm previous findings that cognitive impairment affects about half of all patients with MS [4–6, 15, 35]. Cluster analysis revealed that 46 out of 99 MS male patients and 133 out of 266 MS female patients fit the cluster associated with lower memory functioning. This result shows that grouping MS patients into two clusters—one that refers to lower memory functioning, and the other associated with higher memory functioning—is ideal for further calculating valid discriminant functions. Thus, the cluster of lower memory functioning should be used for further discriminant analysis.

We also confirmed our previous results that a sample of healthy participant might be nonhomogeneous [18, 26]. A control sample of healthy participants must first be separated into clusters prior to performing discriminant analysis. In this study, 9 out of 20 healthy male participants as well as 38 out of 78 healthy female participants fit the cluster associated with lower memory functioning. Thus, the cluster of healthy participants with higher memory functioning should be used for further discriminant analysis.

### 4.3. Separate Discrimination by Readiness to Learn and Ability to Learn

Our results show that first dividing participants and patients by gender and then further dividing them by high versus low recall using standard CVLT-II measures trials 1–5 substantially improves classification of memory impairment in patients with multiple sclerosis. Correct classification reached 100% cases. However, a serious shortcoming still remains regarding the separate assessment of short-term or long-term memory impairment. Impairment of cognitive functioning is correlated with brain atrophy revealed with MRI [7, 8]. Memory has multiple regional atrophy correlates, including deep gray matter [9], cerebral cortex volume [8] and medial temporal lobe volume [10, 11]. Therefore, it is reasonable to hypothesize that MS differentially affects multiple aspects of memory, including attention-mediated or executive aspects such as encoding and retrieval as well as consolidation and perhaps even recognition in some circumstances [10–12]. That is why we hypothesize that, depending on the affected MS brain region, short-term memory, or long-term memory might be impaired predominantly and independently. Therefore, *B*3, reflecting short-term memory, and *B*4, reflecting long-term memory, should be used as separate independent variables.

### 4.4. Discriminant Analysis Using *B*3 or *B*4

Prior to cluster analysis, we modeled each individual learning curve and formed samples of *B*3 and *B*4. Next, we clustered male and female healthy participants and MS patients by *B*3 or *B*4. Then, clusters with high *B*3 and *B*4 values from healthy participants were discriminated against clusters with low *B*3 and *B*4 values from MS patients. Our results show that clusterization of *B*3 and *B*4 samples instead of clusterization standard CVLT-II measures not only gives 100% correct classification as standard Trial 1–Trial 5 measures can do but allows separate assessments of readiness to learn (*B*3) and ability to learn (*B*4) that the standard Trial 1–Trial 5 measures cannot do.

Thus, if an MS male patient achieved *B*3 < 5.84, it suggests an impairment in readiness to learn and, if *B*4 < 12.03, it indicates an impairment in ability to learn. For females, if an MS patient achieved *B*3 < 6.76, it indicates an impairment in readiness to learn and, if *B*4 < 12.79, it indicates an impairment in ability to learn. By adjusting the sample by gender and overall CVLT-II performance, our method of mathematical modeling correctly classified 100% of cases.

## 5. Conclusions

The CVLT-II learning trials 1–5 allow using the learning curve for assessment of possible learning and memory impairment. The CVLT-II provides standardized scores for each of the five learning trials, so that the clinician can compare a patient's raw trials 1–5 scores with standardized scores. However, frequently a patient's raw scores fluctuate due to different factors, so that some of the raw scores are close to the corresponding standardized scores, but others are below normal values. The CVLT-II does not offer any other methods for classifying a patient's learning and memory status based on the learning curve.

The main objective of our research was to illustrate that discriminant analysis is the method of choice, especially if suitable predictor variables are selected. Our results showed that the best predictor variables are coefficients *B*3 and *B*4 of our mathematical model because discriminant functions, calculated separately for *B*3 and *B*4, allow nearly 100% precision of classification. These predictors also allow identification of separate impairment of readiness to learn or ability to learn, or both.

We believe that modeling of the learning curve offers a quick and effective method for assessing overall learning and memory impairment. The use of our mathematical model not only provides clinicians and researchers with additional information not possible with standard measures, it also provides a useful and rapid screening method for detection of early signs of memory impairment in MS patients.

## Figures and Tables

**Table 1:** CVLT-II immediate free recall learning data.

No.	Name of the group	Trial 1 (Mean ± S.E.M.)	Trial 2 (Mean ± S.E.M.)	Trial 3 (Mean ± S.E.M.)	Trial 4 (Mean ± S.E.M.)	Trial 5 (Mean ± S.E.M.)
(1)	Healthy participants, total	7.26 ± 0.21	10.67 ± 0.24	12.22 ± 0.23	13.09 ± 0.21	13.76 ± 0.18
(2)	MS patients, total	6.15 ± 0.10	9.16 ± 0.14	10.46 ± 0.15	11.32 ± 0.15	11.66 ± 0.14
(3)	Healthy participants, males	5.90 ± 0.45	8.75 ± 0.56	10.75 ± 0.42	11.55 ± 0.47	12.50 ± 0.45
(4)	Healthy participants, females	7.60 ± 0.23	11.17 ± 0.24	12.60 ± 0.25	13.49 ± 0.21	14.08 ± 0.18
(5)	MS patients, males	5.65 ± 0.18	8.15 ± 0.25	9.30 ± 0.28	10.17 ± 0.30	10.42 ± 0.30
(6)	MS patients, females	6.33 ± 0.12	9.53 ± 0.16	10.89 ± 0.17	11.74 ± 0.17	12.12 ± 0.16
(7)	Healthy male participants, cluster referring to lower memory functioning	4.44 ± 0.50	7.44 ± 0.53	9.78 ± 0.68	10.33 ± 0.67	11.00 ± 0.60
(8)	Healthy male participants, cluster referring to higher memory functioning	6.82 ± 0.63	9.82 ± 0.81	11.27 ± 0.45	12.36 ± 0.53	13.55 ± 0.34
(9)	MS male patients, cluster referring to lower memory functioning	4.78 ± 0.24	6.41 ± 0.28	7.20 ± 0.28	7.63 ± 0.28	8.02 ± 0.32
(10)	MS male patients, cluster referring to higher memory functioning	6.40 ± 0.21	9.66 ± 0.26	11.13 ± 0.26	12.38 ± 0.25	12.51 ± 0.23
(11)	Healthy female participants, cluster referring to lower memory functioning	6.82 ± 0.29	9.68 ± 0.24	10.92 ± 0.26	12.24 ± 0.25	13.24 ± 0.25
(12)	Healthy female participants, cluster referring to higher memory functioning	8.35 ± 0.31	12.58 ± 0.24	14.20 ± 0.22	14.68 ± 0.20	14.88 ± 0.18
(13)	MS female patients, cluster referring to lower memory functioning	5.32 ± 0.13	7.73 ± 0.15	8.75 ± 0.15	9.68 ± 0.18	10.29 ± 0.17
(14)	MS female patients, cluster referring to higher memory functioning	7.35 ± 0.15	11.34 ± 0.16	13.02 ± 0.13	13.81 ± 0.13	13.95 ± 0.13

**Table 2:** Values of the Model's coefficients for the averaged learning curves.

No	Group	Model's coefficients ± asymptotic standard error			*R* ^2^
		*B*2	*B*3	*B*4	
(1)	All healthy participants	0.65 ± 0.06	7.28 ± 0.13	14.17 ± 0.22	0.9987
(2)	Female healthy participants	0.70 ± 0.07	7.63 ± 0.15	14.38 ± 0.23	0.9982
	Significance level, *P *	0.075	0.0016	0.20	
(3)	Male healthy participants	0.47 ± 0.06	5.90 ± 0.17	13.58 ± 0.48	0.9978
(4)	All MS patients	0.70 ± 0.04	6.16 ± 0.09	12.01 ± 0.13	0.9992
(5)	Female MS patients	0.72 ± 0.04	6.35 ± 0.09	12.44 ± 0.13	0.9993
	Significance level, *P *	0.32	0.006	0.002	
(6)	Male MS patients	0.64 ± 0.06	5.66 ± 0.10	10.86 ± 0.17	0.9987
(7)	Male healthy participants, cluster referring to lower memory functioning using *B*3 with low values	0.4407± 0.0902	3.91 ± 0.30	13.72 ± 0.95	0.9956
	Significance level, *P *	0.68	0.002	0.85	
(8)	Male healthy participants, cluster referring to higher memory functioning using *B*3 with high values	0.5274 ± 0.177	7.5 ± 0.36	13.46 ± 0.85	0.9850
(9)	Male healthy participants, cluster referring to lower memory functioning using *B*4 with low values	1.1353 ± 0.3044	4.38 ± 0.42	9.96 ± 0.37	0.9838
	Significance level, *P *	0.06	0.01	0.0007	
(10)	Male healthy participants, cluster referring to higher memory functioning using *B*4 with high values	0.3361 ± 0.0317	6.39 ± 0.09	15.76 ± 0.49	0.9994
(11)	Male MS patients, cluster referring to lower memory functioning using *B*3 with low values	0.6505 ± 0.0344	4.26 ± 0.06	9.81 ± 0.11	0.9995
	Significance level, *P *	0.73	0.00006	0.002	
(12)	Male MS patients, cluster referring to higher memory functioning using *B*3 with high values	0.6179 ± 0.0821	6.97 ± 0.14	11.85 ± 0.25	0.9971
(13)	Male MS patients, cluster referring to lower memory functioning using *B*4 with low values	0.8192 ± 0.0252	5.17 ± 0.03	8.74 ± 0.03	0.9998
	Significance level, *P *	0.044	0.013	0.0014	
(14)	Male MS patients, cluster referring to higher memory functioning using *B*4 with high values	0.5087 ± 0.1041	6.37 ± 0.28	14.21 ± 0.69	0.9946
(15)	Female healthy participants, cluster referring to lower memory functioning using *B*3 with low values	0.7231 ± 0.0807	5.27 ± 0.24	14.28 ± 0.34	0.9976
	Significance level, *P *	0.75	0.00025	0.72	
(16)	Female healthy participants, cluster referring to higher memory functioning using *B*3 with high values	0.6877 ± 0.064	8.56 ± 0.12	14.43 ± 0.19	0.9984
(17)	Female healthy participants, cluster referring to lower memory functioning using *B*4 with low values	2.0767 ± 3.6190	6.98 ± 1.44	10.81 ± 0.92	0.7277
	Significance level, *P *	0.72	0.66	0.017	
(18)	Female healthy participants, cluster referring to higher memory functioning using *B*4 with high values	0.6616 ± 0.0855	7.67 ± 0.20	14.63 ± 0.33	0.9971
(19)	MS female patients, cluster referring to lower memory functioning using *B*3 with low values	0.6286 ± 0.0614	5.06 ± 0.14	11.62 ± 0.24	0.9984
	Significance level, *P *	0.024	0.00003	0.0011	
(20)	MS female patients, cluster referring to higher memory functioning using *B*3 with high values	0.8507 ± 0.0109	8.08 ± 0.02	13.63 ± 0.02	0.9999
(21)	MS female patients, cluster referring to lower memory functioning using *B*4 with low values	0.8642 ± 0.0812	5.40 ± 0.11	9.88 ± 0.13	0.9981
	Significance level, *P *	0.084	0.0005	0.00003	
(22)	MS female patients, cluster referring to higher memory functioning using *B*4 with high values	0.6541 ± 0.0431	7.01 ± 0.11	14.26 ± 0.17	0.9992
